# Synthesis of a novel nanobioconjugate for targeted photodynamic therapy of colon cancer enhanced with cannabidiol

**DOI:** 10.18632/oncotarget.28171

**Published:** 2022-01-18

**Authors:** Nkune Williams Nkune, Cherie Ann Kruger, Heidi Abrahamse

**Affiliations:** ^1^Laser Research Centre, Faculty of Health Sciences, University of Johannesburg, Doornfontein 2028, South Africa

**Keywords:** cannabidiol, photodynamic therapy, colorectal cancer, nanoparticles, photosensitizer

## Abstract

Photodynamic therapy (PDT) is a promising primary treatment option for colorectal cancer (CRC), however CRC is accelerated by resilient CRC stem-like cells, which decrease its efficacy. In recent years, researchers have shown an emerging interest in the anticancer stem cell effects of cannabidiol (CBD). This study developed a targeted nanobioconjugate for specific ZnPcS4 photosensitizer intracellular accumulation within *in vitro* cultured human CRC cells (CaCo-2) for enhanced PDT primary treatment, as well as limited its secondary spread by combining this treatment with CBD. The final nanobioconjugate (FNBC) was successfully synthesized and characterized using various methods. The cytotoxicity of the FNBC and CBD were tested on CRC cells using laser irradiation at 673 nm with a fluency of 10 J/cm^2^. 24 h post treatment, morphological changes were assessed via microscopy, cell viability was measured using Annexin V-FITC and cellular nuclear DNA was visualized under fluorescent microscopy, following Hoechst staining. FNBC and CBD combinative treatment induced the most significant photodamage, leaving a staggering 6%^***^ viable cells. Overall, through active targeting of CRC cells using the FNBC, the enhanced PDT primary treatment of CRC was achieved, and the combinative treatment with CBD noted significant limitations on its secondary spread.

## INTRODUCTION

Colorectal cancer (CRC) is the third most lethal cancer in the world, which originates from inmost linings of the colon or the rectum following genetic and epigenetic alterations of colonic epithelium into invasive adenocarcinoma [1]. It is a challenging disease to treat due to the high incidence of metastasis, drug resistance, as well as adverse side effects caused by conventional treatments such as surgical excision, chemotherapy, radiation therapy and immunotherapy [2]. Thus, it remains imperative to explore effective therapeutic approaches that may potentially improve prognosis in patients [3]. Photodynamic Therapy (PDT) is a promising non-invasive therapy for premalignant and neoplastic lesions, which is based on three essentials, namely visible light, oxygen, and photosensitizer (PS) [4]. The activation of photosensitizers (PSs) using a suitable wavelength of light, yields cytotoxic reactive oxygen species (ROS) and singlet oxygen, which in turn induce tumour cell death [5]. PDT is comparatively better than conventional treatments, as PSs have high affinity cancer tissues and so exert negligible effects on normal cells due to the enhanced permeability retention (EPR) phenomenon [6]. Thus, when PSs are combined with NPs, their passive uptake in tumour cells is improved via the EPR effective, which is mediated by the leaky tumour vasculature and poor lymphatic drainage of tumour tissues [7–9].

Zinc metallated phthalocyanine (ZnPc) PSs have gained increased popularity in PDT applications due to their high quantum generation, with enhanced tissue penetration depth [10] Studies by Sekhejane reported notable zinc phthalocyanine sulfonated mix (ZnPcS_mix_) PS PDT induced apoptotic cell death of 40% within *in vitro* treated CRC cell lines (DLD-1 and CaCo-2) [11]. However these studies can be improved upon, ZnPcs can be modified with nanocarriers, such as gold nanoparticles (AuNPs), to augment improved passive PS delivery, as well as be conjugated with antibodies to enhance their overall bioavailability in target to tissues [4]. With regards to active targeting, PS nanocarriers can be further modified with targeting entities (e.g., antibodies), which correlate with receptors overexpressed by cancer cells, to enhance their pharmacokinetics and selective targeting affinity [12]. Studies by Danaee noted that CRC tumour receptors tend to specifically over-express the Guanylyl cyclase C (GUCY2C) protein gene and stated that Anti-GCC monoclonal antibodies (mAbs) could be bound to NP-PS molecules, in order to enhance selective active targeting of CRC cells for enhanced PDT treatment [13].

Studies have reported that CRC cells exhibit normal stem like cell properties, called CRC stem cells (CSCs), which promote tumour progression, PDT resistance and metastasis [14]. Thus, this necessitates the development of PDT strategies which allow for annihilation of primary tumours, as well as the inhibition of secondary spread through the induction of anticancer immune reactions which are capable of controlling metastasis [15]. Cannabidiol (CBD) is non-psychoactive compound extracted from the *Cannabis sativa* plant, which is endowed with antiproliferative and pro-apoptotic effects known to impede angiogenesis, cancer cell migration, adhesion and invasion and so can inhibit secondary metastatic cancer spread [16]. Studies by Shams have revealed that the stimulation of the immune system can yield anti-tumour immune responses which can supress metastatic tumour growth [15]. Studies by Jeong investigated the CBD chemo preventive *in vivo* effect in CRC mouse models and noted that through apoptotic cell death pathways it hindered cellular proliferation, as well as generated anti-tumour immune responses post treatment, which controlled CRC metastatic tumour growth and secondary spread [17].

**Figure 1:**
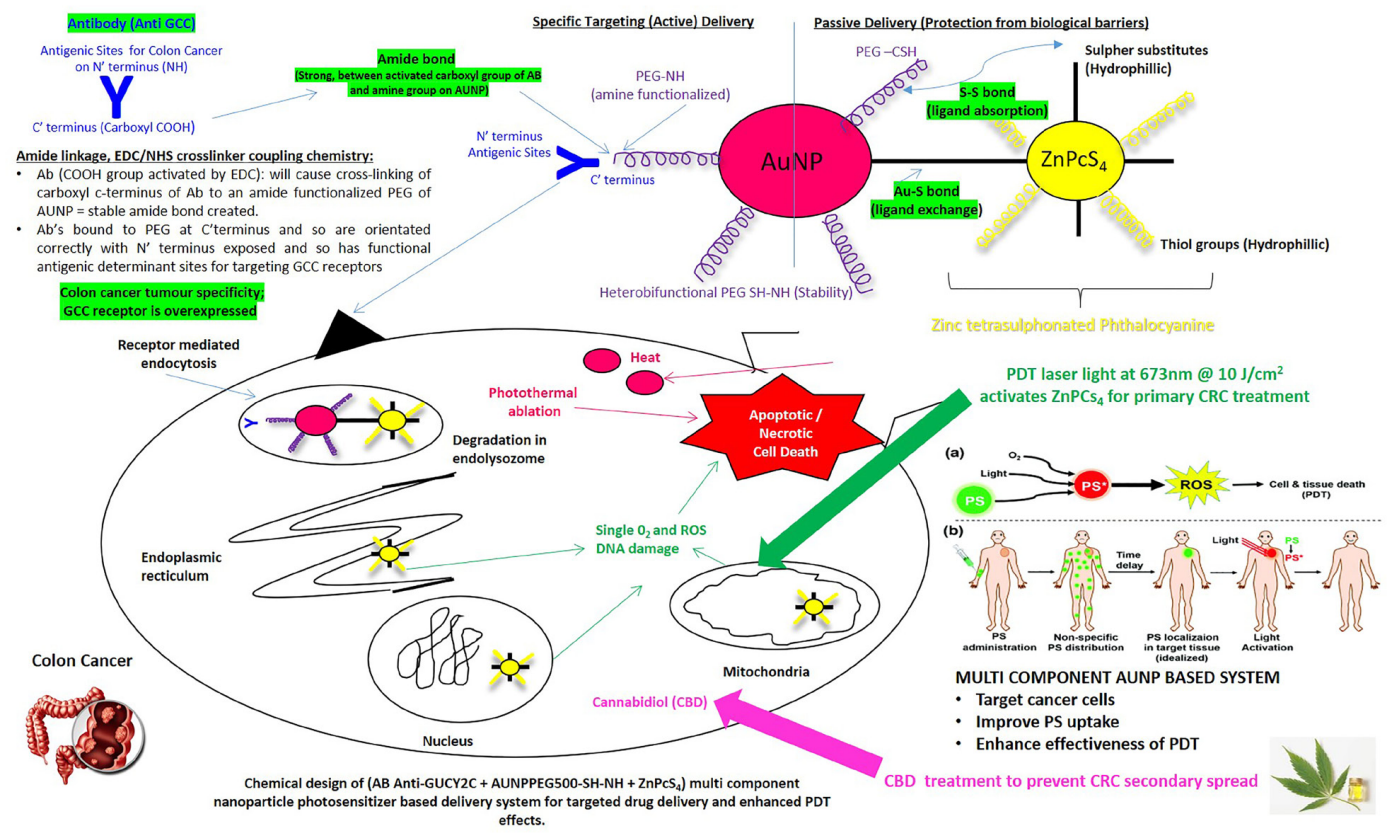
Envisaged structure, bond formation and functioning of the synthesized actively targeted PS final nanobioconjugate: ZnPcS_4_–AuNP-PEG-SH-NH_2_–Anti-GCC Ab (FNBC).

In this study, an efficient cytotoxic oxygen generating hydrophilic tetra sulphonated metal-based PS (Zinc Phthalocyanine Tetrasulfonic Acid, ZnPcS_4_), was bound onto the surface of heterobifunctional pegylated amine stabilized AuNPs, which were conjugated to CRC-specific targeting antibodies (Anti-Guanylate Cyclase Ab: Anti-GCC), to enhance the active PS uptake in CRC cells and so improve the primary PDT treatment of CRC. Moreover, to enhance this CRC targeted primary PDT treatment outcome and hinder secondary reoccurrence, the final targeted PS nanoconjugate was administered with CBD to *in vitro* cultured CRC cells (Figure 1) [18].

## RESULTS

### ZnPcS_4_ photosensitizer and CBD dose response assays

Cellular biological responses were used to determine the lowest possible concentration of ZnPcS_4_ PS or CBD which could be administered to *in vitro* cultured CaCo-2 to yield 50% cell viability, as well as induce a significant amount of cytotoxicity, in order to establish cell death induction potential (ICD_50_), but still allow cellular biological responses post-PDT treatment to be determined. The optimal ICD_50_ dose response concentrations of ZnPcS_4_ and CBD post-PDT treatment that induced approximately 50% cytotoxicity was found to be 0.125 μM ZnPcS_4_ and 1 μM CBD, after observing Trypan Blue cellular viability assay results below.

### Dose response results of ZnPcS_4_

**Figure 2:**
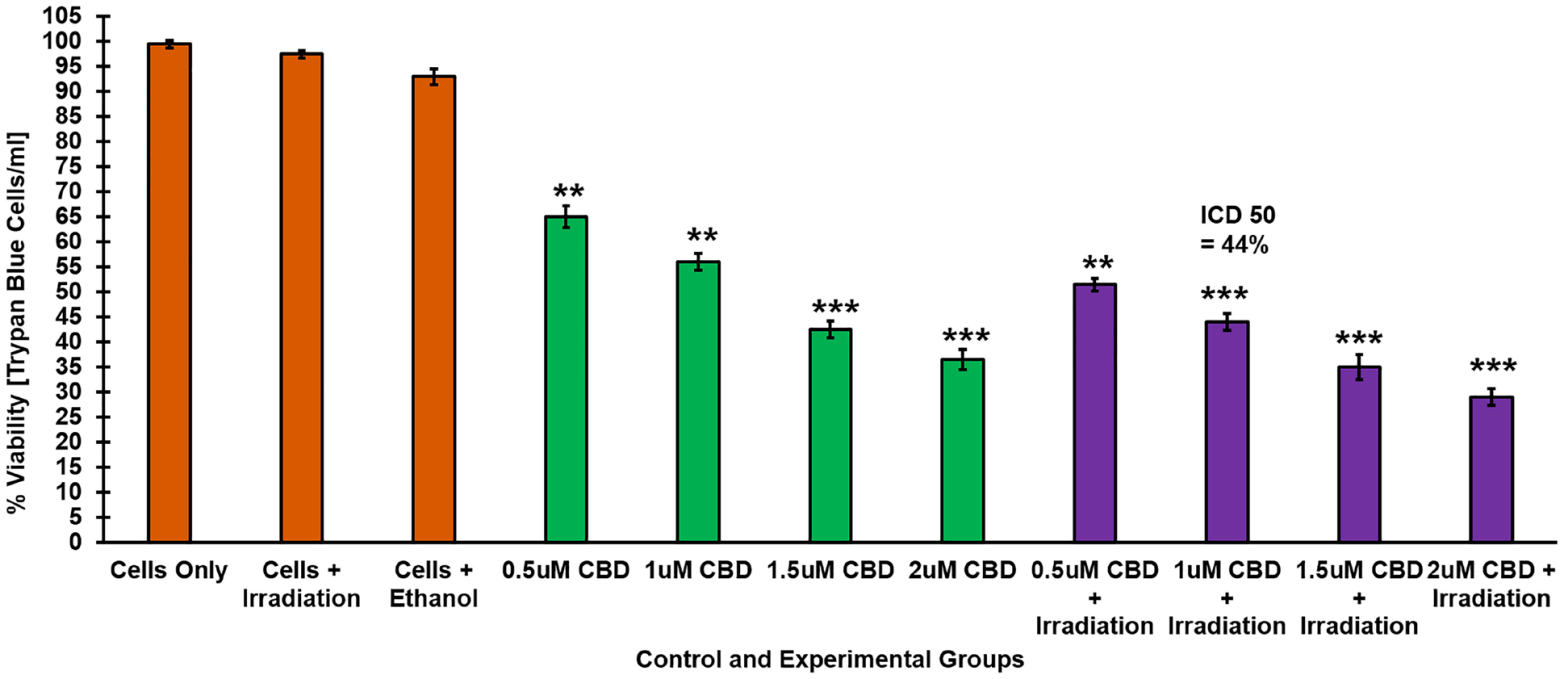
Trypan blue exclusion cell viability assay. Response of CaCo-2 cells treated with varying concentrations of ZnPcS_4_ PS following PDT, demonstrating a decrease in cell viability in a dose dependent manner.

Cellular viability in CaCo-2 cells was evaluated using Trypan blue. Cells incubated with varying concentration of ZnPcS_4_ alone (0.0312, 0.0625, 0.125 and 0.25 μM) showed no significant changes in cellular viability when compared to cells only control exposed to neither light nor PS (Figure 2).

However, PDT treated experimental groups resulted in a significant decrease in cellular viability as compared to untreated cells only control. Within these groups, the cells which received 0.125 μM ZnPcS_4_ PS showed 54%^***^ cell viability and so this concentration was considered ideal concentration for ICD_50_ to be utilized throughout further experimentation.

### Dose response results of CBD

**Figure 3:**
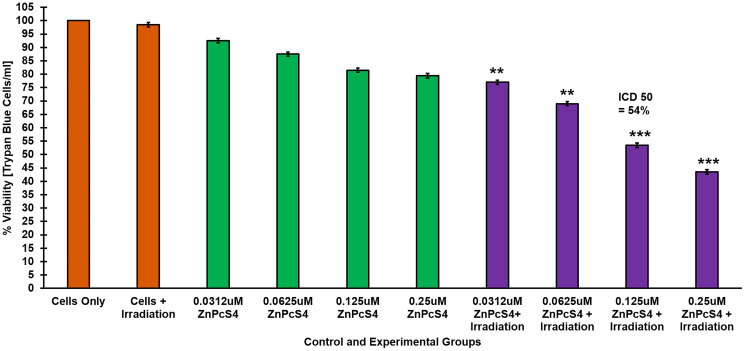
Trypan blue exclusion cell viability assay. Response of CaCo-2 cells treated with varying concentrations of CBD alone and laser irradiation, demonstrating a dose dependent decrease in cell viability.

Cellular viability in CaCo-2 cells was evaluated using Trypan blue. No significant changes in cell viability were noted in neither cells treated with irradiation alone or ethanol alone, when compared to cells only control (Figure 3). However, there was a significant dose decrease in cell viability in cells treated with CBD alone (0.5, 0.1, 1.5 and 2 μM) when compared to cells only control. These findings suggest that CBD alone within the dosing range of 0.5 to 2 μM, demonstrated a dose dependent inhibitory effect of on CRC cells. These findings correlate with studies by Aviello, whereby similar concentrations of CBD, noted a significant decrease in cellular viability via induction of apoptotic cell death [19]. Similarly, a dose dependent decrease in cell viability was noted in irradiated experimental groups incubated with CBD at concentration of 0.5 to 2 μM. Within these experimental groups, 44%^***^ of the cells were found be viable when 1 μM CBD was administered to the cells and so 1 μM was considered the ideal ICD_50_ concentration for CBD plus irradiation to be utilized throughout further experimentation.

### Molecular characterization of the final nanobioconjugate (ZnPcS_4_ – AuNP – Anti-GCC)

UV spectra and FTIR results established that ZnPcS_4_ PS was successfully bound onto the AuNPPEG’s surface, with strong Au-S and weak di-sulphide bonds, via ligand absorption and exchange methods. Furthermore, these assays confirmed that the Anti-GCC mAbs were successfully conjugated on the amine functionalized AuNP-PEG-SH-NH_2_ via amide bonding. ZP results noted that the final nanobioconjugate (FNBC) had an average diameter of 57.18 ± 3.04 nm, suggesting it was of an acceptable nanoscale size for active drug carrying that could pass through cellular membranes [20]. Furthermore, the FNBC had a positive ZP value of 36.5 ± 2.6 mV, noting that it was highly stable, suggesting that within tumour microenvironments it should remain stable and retained easily [21, 22]. DLS PDI value of 0.353 results found the FNBC to be monodispersed, spherical in shape with little aggregation [21]. Overall, these results noted that the ZnPcS_4_ PS, AuNP-PEG-SH-NH_2_’s and Anti-GCC Ab were successfully conjugated to one another to form one single molecule [21]. Moreover, within UV spectra assays FNBC confirmed UV-Vis absorption Q bands within the 520, 634 and 674 nm wavelength regions, showing it retained its photodynamic and photothermal properties and remained photostable.

### UV-Visible spectroscopy

**Figure 4:**
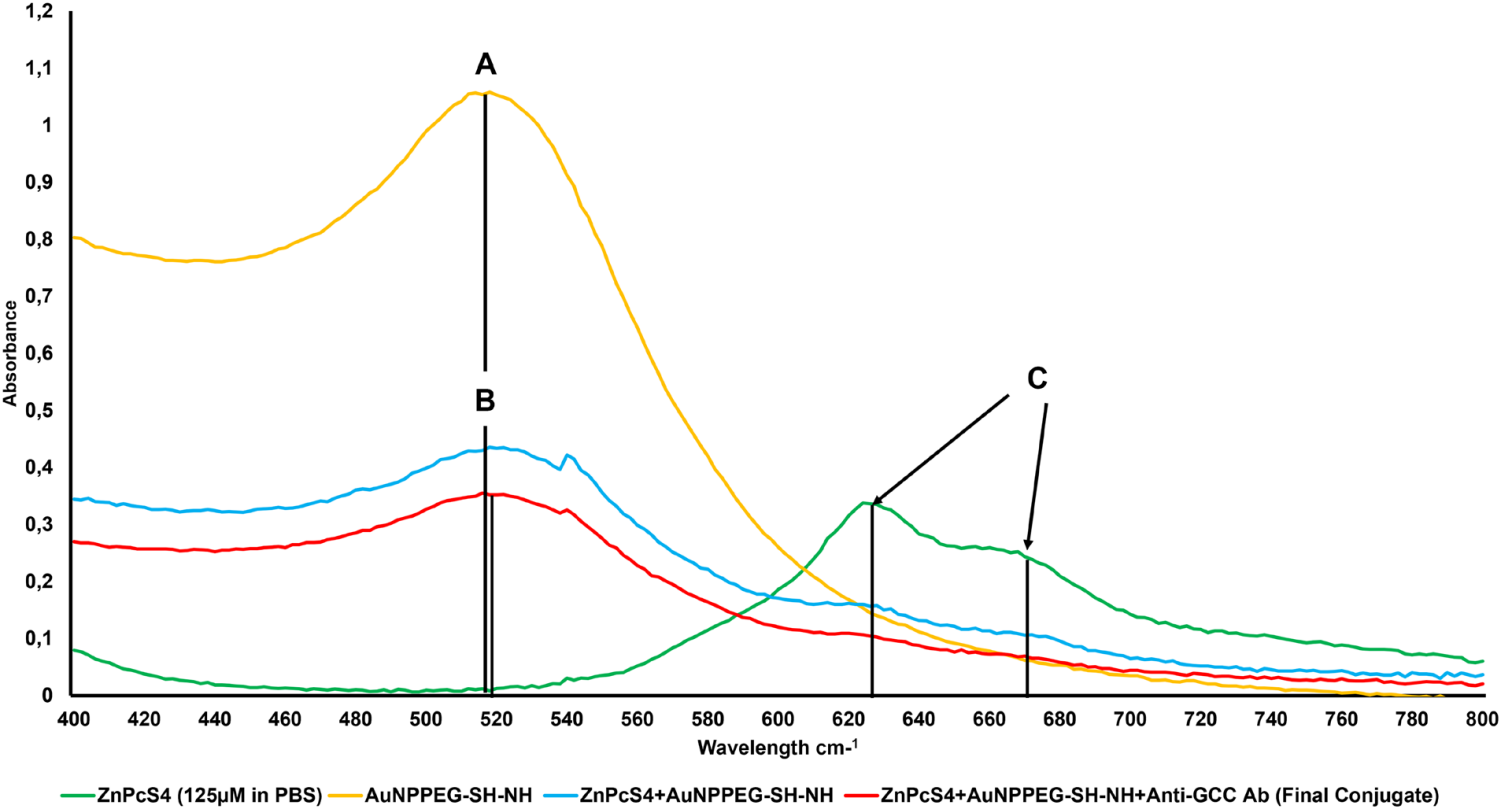
Spectral analysis of the FNBC and its components within the 400 to 800 nm spectral range. (**A**) AuNPEG500-CSH-NH 520 nm 2.85 × 10^15^ particles/ml, (**B**) FNBC 520 nm 0.95 × 10^15^ AuNP particles/ml (2.99 fold fall) and (**C**) ZnPcS_4_ 634 and 674 nm 125 μm in PBS, whereas th FNBC is 35 μM ZnPcS_4_ concentration (3.54 fold fall).

The spectral analysis of the FNBC were recorded using spectrum/purity scan mode within the 400 to 800 nm spectral range. Figure 4 shows distinctive absorbance spectra of ZnPcS_4_, two major Q bands of emission at 634 and 674 nm and a typical AuNPEG-SH-NH_2_ absorption spectrum at 520 nm correlated with 2.85 × 10^15^ particles/ml [23]. Within the FNBC spectra, ZnPcS_4_ and AuNPEG-SH-NH_2_ peaks were notable, indicating that both compounds had bound, however lowered in concentration when compared to each component individually. Therefore, by comparing the absorbance fold falls of 125 μM ZnPcS_4_ at 673 nm and AuNPEG-SH-NH_2_ (2.85 × 10^15^ particles/ml) at 520 nm, to the absorbance peak values exhibited by the FNBC within these ranges, it was concluded that the FNBC (ZnPcS_4_–AuNP-PEG-SH-NH_2_–Anti-GCC Ab) consisted of 0.95 × 10^15^ particles/ml AuNPEG-SH-NH_2_ bound to 35 μM of ZnPcS_4_.

The FNBC obtained a distinct ZnPcS_4_ absorption peak at 673 nm, this was an indication that ZnPcS_4_ photochemical properties pivotal for PDT-mediated ROS and singlet oxygen yield remained intact after conjugation (Figure 4) [23]. Furthermore, this FNBC obtained a prominent absorption peak of AuNPEG-SH-NH_2_ at 520 nm, suggesting that it can exert appreciable photothermal and photodynamic effects [24]. Furthermore, the absorption peaks obtained at 520 and 673 nm by the FNBC in comparison to ZnPcS_4_ and AuNPEG-SH-NH_2_ control peaks, slightly broadened and still appeared sharp and smooth, suggesting that an anticipated size distribution with minor aggregation was attained (Figure 4) [25]. However, with the absorption peaks of the FNBC a slightly broadening could be noticed, this is suggestive of a definitive bonding between all the chemical components, due to the increment in the molecular size [4]. Lastly, the FNBC noted a minor resonance peak position shift at 520 nm (AuNP band), suggesting that the Anti-GCC Ab and ZnPcS_4_ were successfully bound to its surface [26].

**Figure 5:**
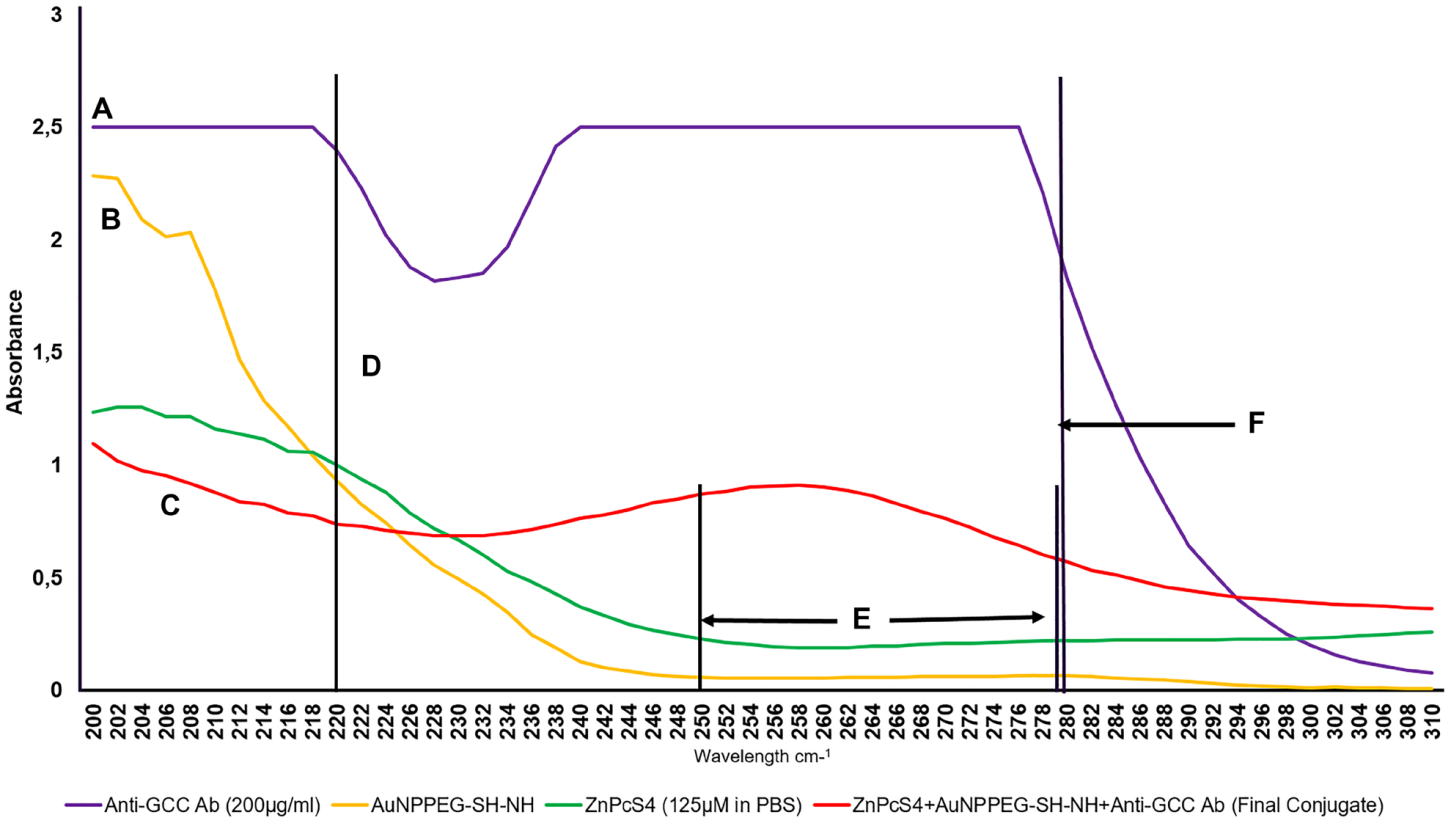
UV-Visible protein direct absorption spectra of the FNBC and controls within 200 to 310 nm spectral region. (**A**) Amine NH_2_ 195 nm group on Ab N’terminal, (**B**) Amine NH_2_ 195 nm group on functionalized AuNPPEG-NH_2_, (**C**) Amine NH_2_ 195 nm group on final conjugate Ab N’terminal (Ab functional), (**D**) Peptide/amide bond 220 nm of FNBC (Functionalized AuNPPEG-NH_2_ lost amine group to bond with carboxyl C-terminus of Ab and form a peptide bond), (**E**) Di-sulphide bond 250 to 280 nm (FNBC higher UV-Vis spectral absorption than AuNPPEG500-SH-NH_2_ and ZnPcS_4_ à ligand absorption between the sulphonated PS and AuNPPEG500-SH, creating di-sulphide bond) and (**F**) Anti - GCC Ab Protein 280 nm 200 μg/ml (6.47 times higher absorbance than FNBC – suggesting final concentration of Ab on conjugated molecule is 30.91 μg/ml).

The UV-Visible 280 nm protein spectral region of the FNBC noted a 6.47 absorbance fall fold when linked to the original 200 μg/ml Anti-GCC Ab concentration (Figure 5). This confirms that the FNBC consistent then of 30.91 μg/ml of bound Anti-GCC Ab. Standard literature states that amine functional groups (NH_2_) display a prominent 195 nm peak, whereas a strong 220 nm absorption peak is indicative of amide peptide bond (CO-NH) formation when the condensation of an amine with a carboxylic acid has occurred [25]. AuNPEG-SH-NH_2_ control obtained a notable 195 nm absorbance, which is anticipated since it was amine functionalized. Similarly, with Anti-GCC control, due to the amine groups on its N-terminal. However, the FNBC noted a reduced amine functional group (NH_2_) absorption peak (195 nm), in comparison to that of the AuNPEG-SH-NH_2_ control, confirming the condensation of the AuNP’s amine functional group with the activated C-terminus of the Abs carboxylic group to form an amide bond (CO-NH). This final amide bond (CO-NH) was further verified by noting a prominent 220 nm absorption peak within the FNBC wavelengths spectra. Moreover, since the FNBC still retained an amine functional peak (195 nm), this was conclusive that AuNPEG-SH-NH formed an amide bond with the C-terminus of Anti-GCC Ab leaving its antigen binding site (N-terminus) primed for active PS delivery. Lastly, in comparison to AuNPEG-SH and ZnPCS_4_ controls the FNBC obtained a detectable absorption peak within the 250 to 280 nm range suggesting that sulphonated ZnPcS_4_ PS and AuNPEG-SH could have formed di-sulphide bond via ligand absorption [27].

Lastly, these results distinguished that the FNBC contained 0.95 × 10^15^ AuNPPEG-SH-NH_2_ particles/ml which were successfully conjugated to 30.91 μg/ml of Anti-GCC Ab and 35 μM of ZnPcS_4_ in 0.001 M PBS (w/v). These findings meant that the FNBC required a dilution correction factor to bring the ZnPcS_4_ PS concentration down to recommended ICD_50_ dose concentration of 0.125 μM, when being added to the various control and experimental culture plates. After diluting the experimental synthesized FNBC, using a 280-fold dilution factor to contain 0.125 μM ZnPcS_4_ PS, it must be noted that this also affected the final concentration of the AuNPPEG-SH-NH_2_ and Anti-GCC Ab. Therefore, within the FNBC PDT response assays, within *in vitro* cultured CRC, culture plates received 0.11 μg/ml Anti-GCC Ab, which was successfully bound to 3.39 × 10^12^ AuNPPEG-SH-NH_2_ particles/ml conjugated to a 0.125 μM of ZnPcS_4_ in 0.001 M PBS (w/v).

### Fourier transform infrared (FTIR) spectroscopic analysis

**Figure 6:**
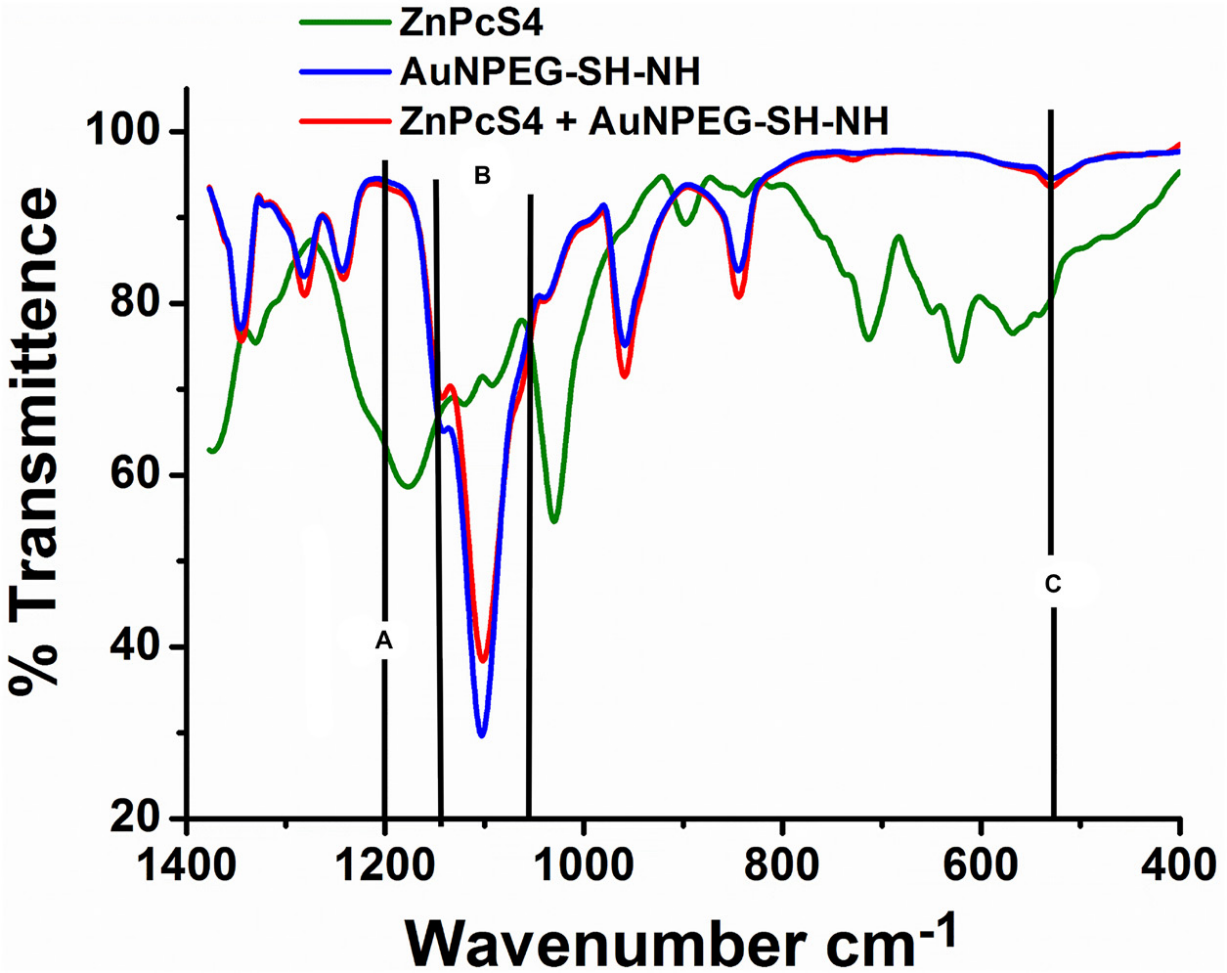
FTIR spectral analysis confirming ligand exchange (Au-S) and absorption (S-S) bond formation between AuNPPEG-SH-NH_2_ and ZnPcS_4_ within the FNBC. (**A**) C-S (1200 cm^–1^ Sharp): Au-S ligand exchange bond bond formed as ZnPcS_4_ PS lost its C-S groups to form a Au-S bond with the AuNPs, (**B**) C-S (1050–1200 cm^–1^ Stretch): Au-S ligand exchange bond formed as AuNPs PS lost its C-S groups to form a Au-S bond with ZnPcS_4_ PS and (**C**) S-S (500–540 cm^–1^ Bend): Disulphide (SS) bond formed due to ligand absorption between the sulphonated ZnPcS_4_ PS and AuNPPEG5000-SH.

FTIR spectroscopy is a widely used technique in structural identification, and can be used for quantitative, as well as qualitative analysis of the FNBC [27]. Spectral analysis of AuNPEG-SH-NH_2_–ZnPcS_4_ revealed a 1050–1200 cm^−1^ C-S stretch shift, suggesting that the AuNPEG-SH-NH_2_ dropped their C-S group to bind with ZnPcS_4_ and so form an Au-S ligand exchange bond (Figure 6) [28, 29].

This Au-S ligand exchange bond formation was additionally confirmed since the spectra of AuNPEG-SH-NH_2_–ZnPcS_4_ noted a loss of a 1200 cm^−1^ C-S sharp band, when compared to ZnPcS_4_ alone, suggesting ZnPcS_4_ lost its C-S group to bind with AuNPEG-SH-NH_2_. Finally, the spectra of AuNPEG-SH-NH_2_–ZnPcS_4_ exhibited a 500–540 cm^−1^ sharp S-S band indicating that ligand absorption had occurred between the sulphonated ZnPcS_4_ PS and AuNPEG-SH-NH_2_ to create a weak di-sulphide bond.

**Figure 7:**
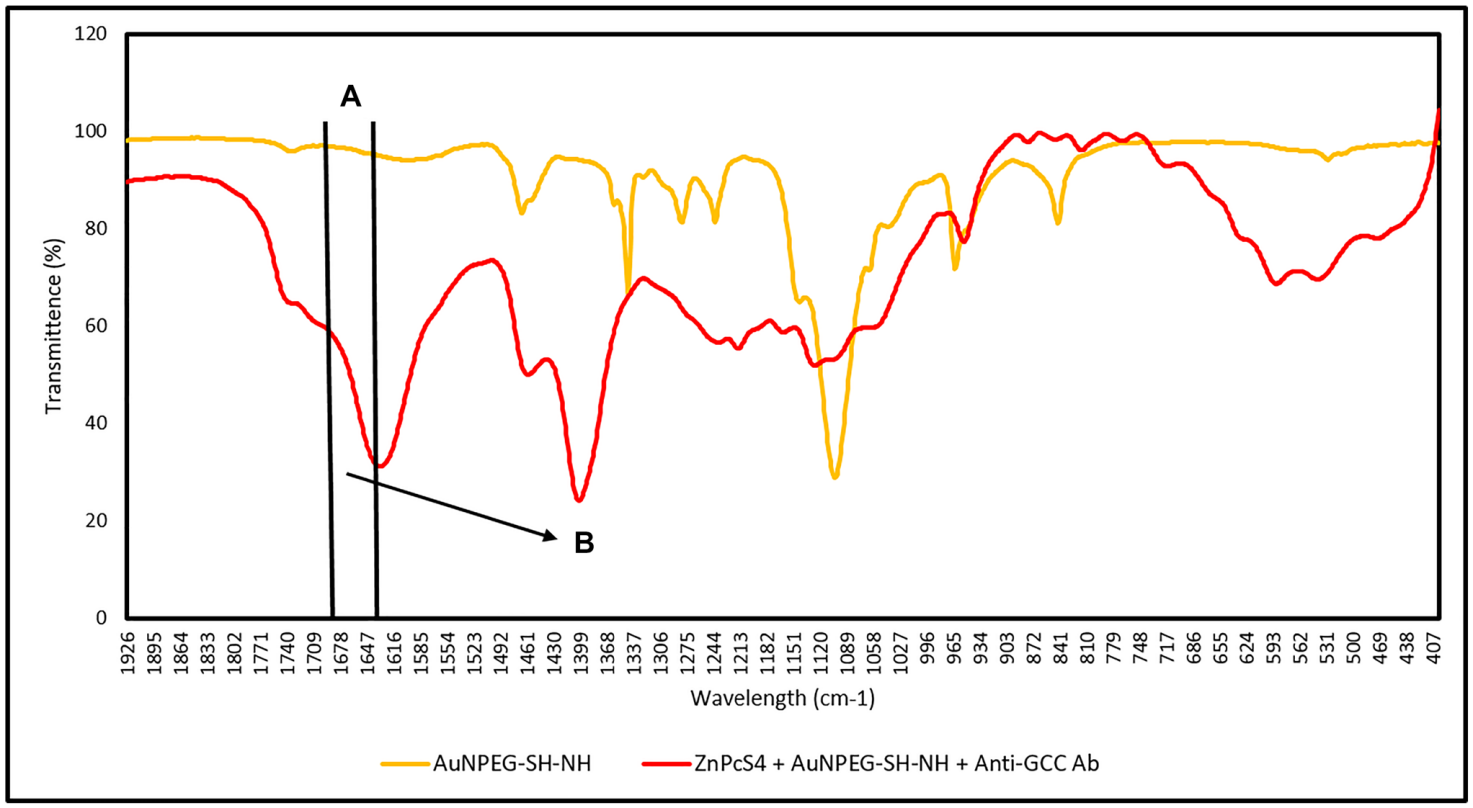
FTIR spectral analysis confirming FNBC amide (CO-NH) bond formation between AuNP-PEG-SH-NH_2_ and the activated carboxylic group on the Anti-GCC Ab C-terminus. (**A**) C = O (1680–1630 cm^–1^ Stretch): Amide peptide bond (CO-NH) formed between amine (NH) AuNP functional group and activated carboxylic group on the C-terminus of the Ab and (**B**) N-H (1640–1650 cm^–1^ Bend): Secondary amide (NH) bond formed between amine (NH) AuNP functional group and activated carboxylic group on the c’ terminus of the Ab.

Additionally, the FNBC amide bond formation was confirmed by comparing its FTIR spectra of AuNPEG-SH-NH alone (Figure 7). The presence of the 1680–1630 cm^−1^ C = O stretch band in the FNBC FTIR spectra confirms amide bond formation alone, but the 1640–1650 cm^−1^ N = H band is not clearly evident, when compared to the AuNPEG-SH-NH_2_ alone, this suggests that strong (CO-NH) primary and secondary amide bonds had formed between the AuNPs amine (NH) functionalized group and the activated Anti-GCC Ab C-terminus [29].

### Dynamic light scattering (DLS) and zeta potential (ZP) analysis

Table 1 shows the DLS and ZP results. The DLS results noted the FNBC produced one single major peak that was narrow width with no additional smaller side peaks, suggesting it was homogenously pure, spherical shaped with no aggregation. The FNBC noted a mean Z-average of 57.18 ± 3.04 nm, epitomizing a desirable active nanodrug carrier [30]. The FNBC noted a PDI value of 0.353, suggesting it was monodispersed and consisted of mostly singular sized particles. The ZP value of the FNBC was 36.5 ± 2.6 mV, suggesting that it is highly stable with a slightly positively charge and will remain stable under *in vivo* conditions, as well as be retained within cancer tumour cells more selectively for a longer period of time [20, 30].

**Table 1:** DLS and ZP results for characterization of final NP PS drug delivery system

Sample	DLS Mean Z-Average Diameter (nm)	Polydispersity Index (PDI)	Zeta Potential (mV)
AuNP	11.78 ± 0.966	0.351 (Monodisperse)	
ZnPcS_4_	18.15 ± 1.44	0.107 (Monodisperse)	
Anticipated average ZnPcS_4_ - AuNP	48.08 ± 1.20		
Result mean ZnPcS_4_ - AuNP	44.57 ± 0.564	0.250 (Monodisperse)	
Anti-GCC Ab	5.21 ± 0.51	0.400 (Monodisperse)	
			
Anticipated average ZnPcS_4_ - AuNP - Anti-GCC Ab	54.99 ± 0.537		
			
Result mean of FNBC	57.18 ± 3.04	0.353 (Monodisperse)	36.5 ± 2.6 (Highly stable)

### Subcellular localization

**Figure 8:**
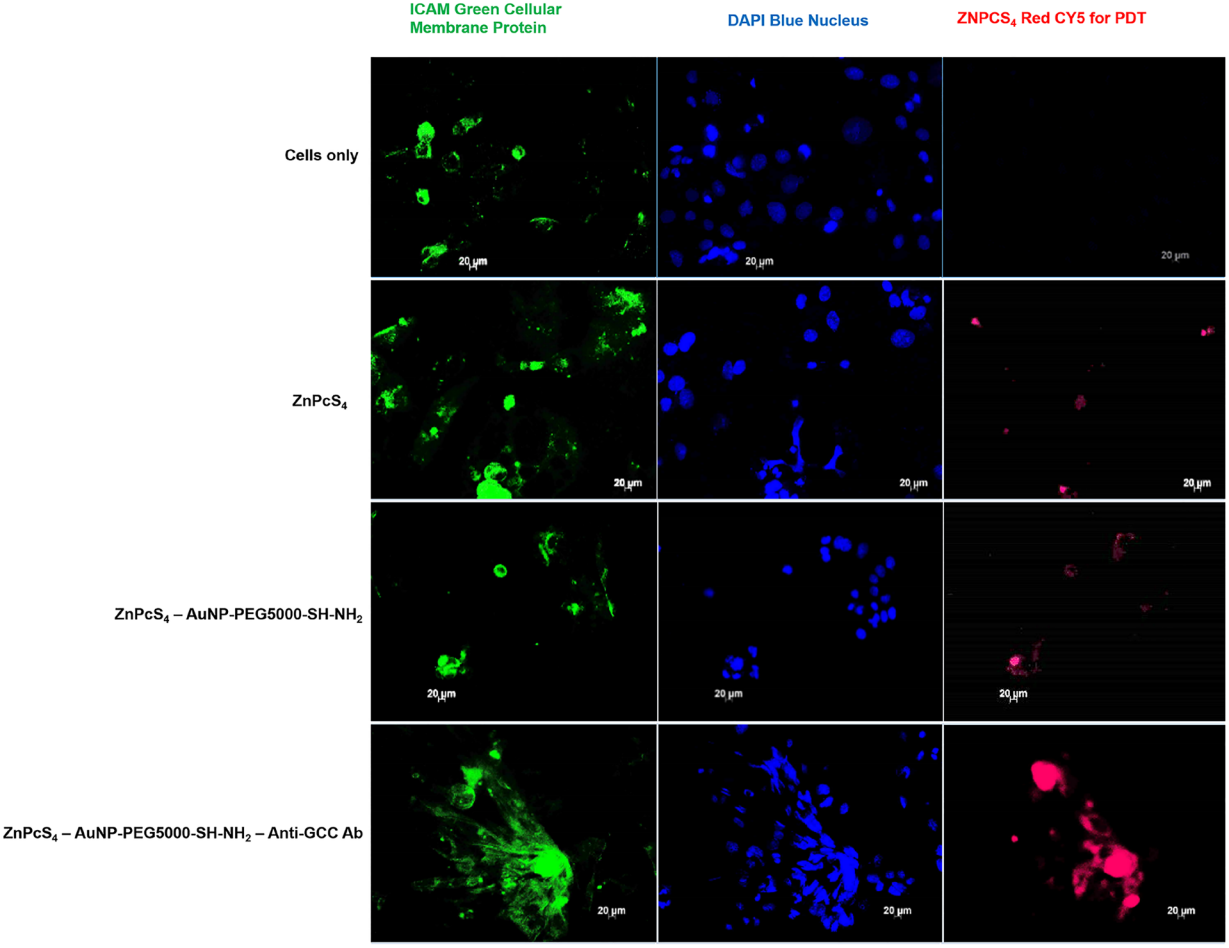
Subcellular localization comparison of ZnPcS_4_ PS uptake in CaCo-2 cells, treated with ZnPcS_4_ PS alone, PS-AuNP and FNBC after 24 h of incubation. Images in the first column show ICAM-stained cellular membrane proteins (Green), images in the second column show DAPI-stained nuclei (Blue) and images in the third column show ZnPcS_4_ red fluorescence (RED).

Subcellular studies reported an immensely improved accumulation of PS in CRC cells treated with FNBC, as compared to PS (ZnPcS_4_) and PS-AuNP (ZnPcS_4_–AuNP), which was indicated by an increased red intensity in cells (Figure 8).

These results indicate that the targeting affinity of the FNBC mediated by Anti-GCC Ab, was specific and efficient within *in vitro* cultured CRC cells membrane proteins and nuclei. Moreover, this specific active targeting FNBC improved PS subcellular localization within CRC cells far more superior than when compared to control groups, suggesting that the FNBC possibly could enhance PDT treatment out comes due to this enhanced PS retention and concentrated absorption.

### Morphological analysis

**Figure 9:**
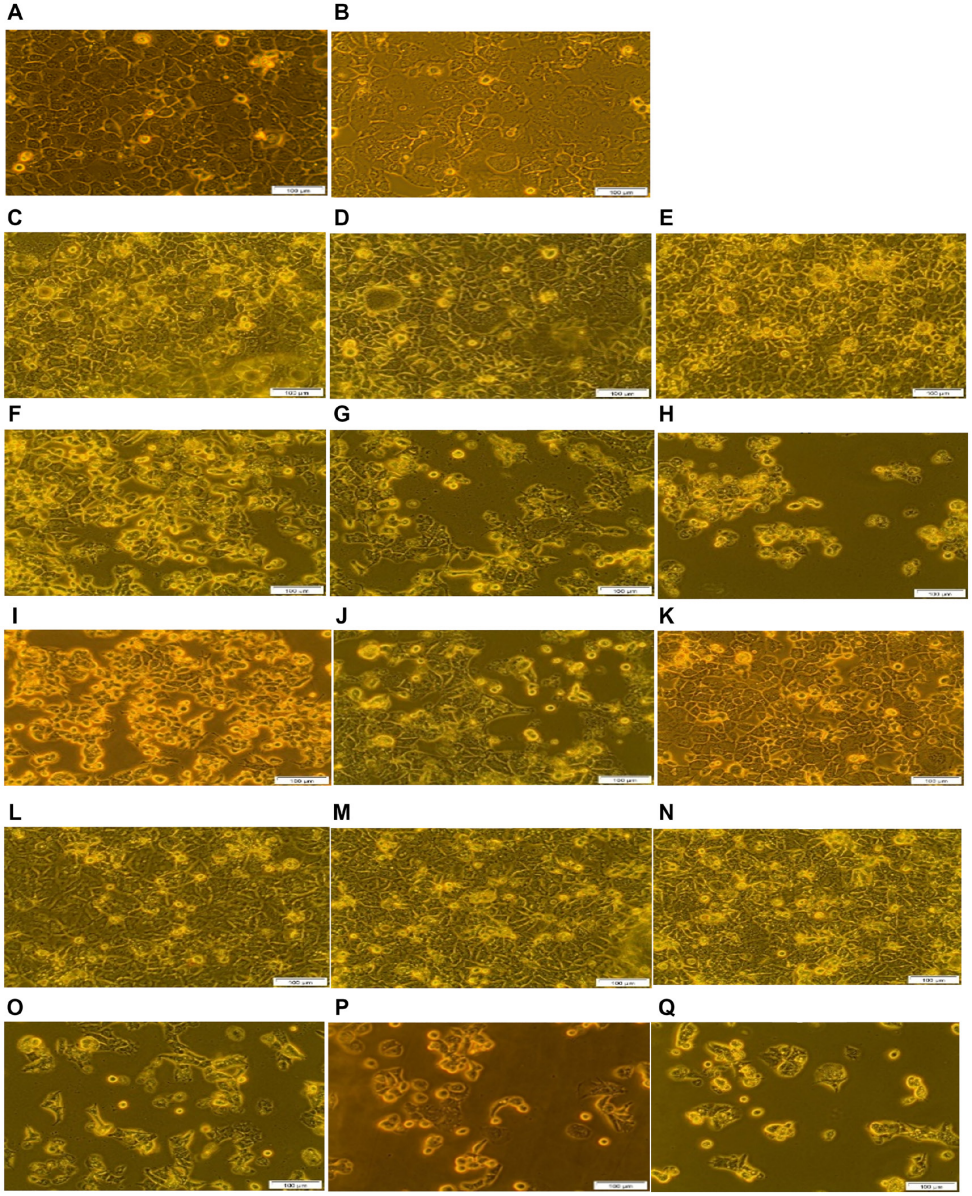
Morphological analysis of the FNBC PDT response assays with and without CBD treatment. (**A**) Cells only, (**B**) Cells + Irradiation (10 J/cm^2^), (**C**) Cells + 0.125 μM ZnPcS_4_, (**D**) Cells + 0.125 μM ZnPcS_4_ – AuNP-PEG5000-SH- NH_2_, (**E**) Cells + 0.125 μM ZnPcS_4_ – AuNP-PEG5000-SH-NH_2_ – Anti-GCC Ab, (**F**) Cells + 0.125 μM ZnPcS_4_ + Irradiation, (**G**) Cells + 0.125 μM ZnPcS_4_ – AuNP-PEG5000-SH- NH_2_ + Irradiation, (**H**) Cells + 0.125 μM ZnPcS_4_ – AuNP-PEG5000-SH- NH_2_ – Anti-GCC Ab + Irradiation, (**I**) Cells + 1 uM CBD, (**J**) Cells + 1 uM CBD + Irradiation, (**K**) Cells + ethanol, (**L**) Cells + 0.125 μM ZnPcS_4_ + 1 uM CBD, (**M**) Cells + 0.125 μM ZnPcS_4_ – AuNP-PEG5000-SH- NH_2_ + 1 uM CBD, (**N**) Cells + 0.125 μM ZnPcS_4_ – AuNP-PEG5000-SH- NH_2_ – Anti-GCC Ab + 1 uM CBD, (**O**) Cells + 0.125 μM ZnPcS_4_ + 1 uM CBD + Irradiation, (**P**) Cells + 0.125 μM ZnPcS_4_ – AuNP-PEG5000-SH- NH_2_ + 1 uM CBD + Irradiation and (**Q**) Cells + 0.125 μM ZnPcS_4_ – AuNP-PEG5000-SH- NH_2_ – Anti-GCC Ab + 1 uM CBD + Irradiation.

Changes in cellular morphological were observed using light microscopy. No morphological changes were noted between cells which received laser treatment alone and cells treated with either PS or its conjugates alone, when compared to the cells only control (Figure 9). These findings suggest that the application of PS or its conjugates alone induced no phototoxicity or dark toxicity. However, free-floating cells, detachment, and loss of shape were observed for the cells treated with ZnPcS_4_ PS, ZnPcS_4_ PS-AuNP or FNBC in the presensce of laser light irradiation. Within these groups, the most significant damage was noted in cells treated with FNBC, whereby the cells appeared to be completely rounded up and detached. It can be concluded that when AuNP-PSs are incorporated with active targeting moieties (e.g., mAbs) this can enhance ZnPcS_4_ PS cellular uptake, resulting in improved PDT efficacy.

In Figure 9, cells treated with ethanol alone noted no significant morphological changes, suggesting that ethanol concentration in which CBD was solubilized exhibited no cytotoxicity. Moreover, cells which received either CBD alone or with irradiation, demonstrated significant morphological alterations. These results correlate with studies conducted by Honarmand which considered CBD as an anticancer medicine, since it triggered apoptosis and decreased cellular pleomorphism of CRC cells [31]. These findings suggest that CBD alone exerts a significant inhibitory effect in cancer cells and irradiation elicited negligible effects. Non-irradiated cells treated with CBD coupled with ZnPcS_4_ PS, ZnPcS_4_ PS-AuNP and FNBC noted slightly significant changes in morphology which was attributed to CBD since the PS exhibited no dark toxicity. Lastly, PDT treated groups coupled with CBD reported severely significant morphological changes in relation to loss of attachment characteristics, nuclear impairment and rounding up, in comparison with the same groups without CBD. In this regard, FNBC coupled with CBD induced the most severe morphological destruction due to enhanced intracellular accumulation of PS facilitated by the Ab, as well as photothermal destruction elicited by the AuNP and the notable cytotoxicity of CBD.

### Flow cytometry: cell viability analysis

**Figure 10:**
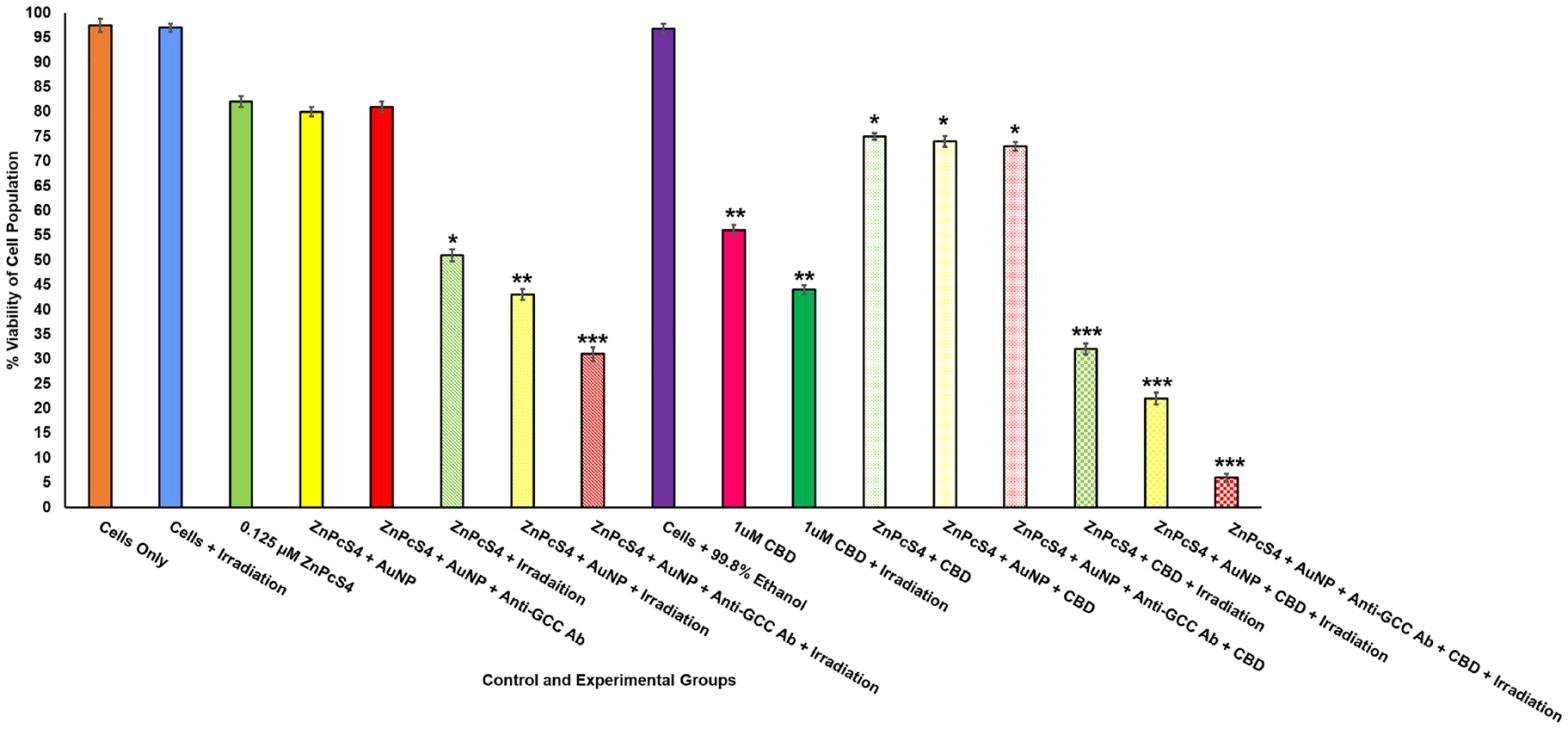
Annexin V-FITC flow cytometry cell viability of FNBC with and without CBD treatment. Significant differences as compared to cells only control are shown as (*P* < 0.05^*^, *P* < 0.01^**^ or *P* < 0.001^***^). 24 h post irradiation , there was a significant decrease cell viability in cells treated with FNBC with and without CBD treatment.

Changes in CRC cell viability of various controls and PDT experimental groups were assessed 24 h post treatment and were compared to the cells only control (Figure 10). No significant differences were seen in cells treated with laser irradiation alone, ZnPcS_4_ PS, ZnPcS_4_ PS-AuNP and FNBC alone.

PDT irradiated experimental groups, which received 0.125 μM ZnPcS_4_ PS alone, noted a significant decrease in cellular viability of 51%^*^, whereas cells which received 0.125 μM ZnPcS_4_-AuNP noted a further significant decrease in cellular viability of 43%^**^. However, PDT irradiated experimental groups, which received the FNBC noted the most significant decrease in cellular viability of 31%^***^. Since second-generation PSs (such as ZnPcS_4_) when administered alone are hydrophobic and tend to aggregate under physiological conditions, this drastically hinders their PDT cytotoxic species formation, due to their limited uptake in tumour cells, which can affect their overall treatment outcomes [9]. This could perhaps explain why the 0.125 μM ZnPcS_4_ PS PDT treatment did not exhibit more significant cellular destruction (cellular viability 51%^*^), as when compared to the same group, which received 0.125 μM ZnPcS_4_ PS -AuNP (cellular viability 43%^**^). Furthermore, studies by Hong have also stated that when PSs are combined with AuNPs, their passive uptake and solubility in tumour cells is enhanced via the EPR effect and so more favourable PDT induced cell death outcomes are found [7]. Additionally, AuNPs also to enhance PDT cellular destruction outcomes, as when they are activated with laser irradiation they produce photothermal energy [9]. Thus the possible improved PS FNBC uptake and photothermal energy probably attributed to the most significant destruction/lowering of cellular viability to 43%^**^ being noted for the PDT irradiated experimental groups which received 0.125 μM ZnPcS_4_ PS-AuNP [9].

However, the most favourably significant and cytotoxic form of PDT induced tumour cell death was found within the PDT experimental groups, which received 0.125 μM FNBC and irradiation, whereby the most cellular viability was only found to be 31%^***^. This highly significant decrease in cellular viability was attributed to the fact that the FNBC contained an actively selective CRC targeting mAb, which promoted ZnPcS_4_ PS localization in CRC cells and so improved overall treatment outcomes, with significant cell death induction. These findings can be supported by Kruger and Abrahamse, whereby the most significant PDT cellular destruction was noted when bound AuNP-PSs were conjugated to targeting mAbs, since their specific and active uptake was localized and with additional photothermal contributions this form of targeted PDT was found to be the most effective in cancer cell obliteration [6].

In relation to control groups of CBD, a significant decrease in cell viability was observed in cells treated with 1 μM CBD alone (56%^**^), suggesting that CBD treatment alone is a highly effective antiproliferative and chemotherapeutic compound. Studies performed by Aviello noted similar findings within CRC *in vitro* treated cells treated with comparable concentrations of CBD alone and noted that CBD reduced cellular proliferation, though anti-tumour immune responses and apoptotic cell death post treatment [24]. Furthermore, results coincide with studies by Raup-Konsavage who reported that CBD drastically decreased cellular viability of various CRC cell lines below 50% [32]. Comparatively CRC control groups which received 1 μM CBD and laser irradiation noted a similarly significant decrease of cellular viability of 44%^**^. Since, there was no statistically significant decrease comparison between the above two discussed control groups, it can be stated that laser irradiation has no effect on the treatment outcome effects of CBD.

Non-irradiated control groups, which received 0.125 μM ZnPcS_4_ PS, 0.125 μM ZnPcS_4_ PS-AuNP and FNBC, plus with 1 μM CBD, noted an average significant decreases in cellular viability of ± 74%^*^. These findings suggest that ZnPcS_4_ PS alone, in conjugation with AuNPs or when finally bound as a FNBC exhibited no dark cytotoxicity and remained in active. Thus, without PDT activation the significant decrease in cellular viability had to be attributed to the 1 μM CBD administration alone, as observed within the previously discussed CBD control groups. The reasoning for perhaps less significant decreases of cellular viability in the non-irradiated control groups, which received 0.125 μM ZnPcS_4_ PS, 0.125 μM ZnPcS_4_ PS-AuNP or FNBC, plus with 1 μM CBD, in comparison to control groups treated with 1 μM CBD with and without laser irradiation, is probably attributed to the fact that the additional PS and conjugate dosing could have diluted the CBD potency.

PDT irradiated experimental groups, which received 0.125 μM ZnPcS_4_ PS, 0.125 μM ZnPcS_4_ PS-AuNP, or FNBC, all with 1 μM CBD, noted extremely significant decreases in cellular viability, in comparison to the same control PDT groups without CBD treatment. PDT irradiated experimental groups, which received 0.125 μM ZnPcS_4_ PS, plus 1 μM CBD reported a significant 32%^***^ decrease in cellular viability, whereas PDT irradiated experimental groups, which received 0.125 μM ZnPcS_4_ PS-AuNP, plus 1 μM CBD reported a significant 12%^***^ decrease in cellular viability. This significantly improved cellular viability destruction in PDT irradiated experimental groups, which received 0.125 μM ZnPcS_4_ PS-AuNP, plus CBD could be attributed to the improved passive uptake and solubilization of the ZnPcS_4_ PS, due to the AuNP conjugated carrier, as well as the photothermal contributions it made to the overall treatment [7]. Additionally, the CBD itself most definitely improved upon the PDT induced cellular death mechanisms, especially when the above results are compared to the same PDT irradiated experimental groups, which did not receive CBD.

However, the most favourably significant results were the PDT irradiated experimental groups, which received the FNBC plus 1 μM CBD, whereby only 6%^***^ of cells were found to be viable. When this result was compared to the PDT irradiated experimental groups, which received the FNBC only and a significant decrease in cellular viability was found to be 31%^***^, the significant final killing off of the CRC cells can most definitely be attributed to the addition of CBD. These findings can be supported by Kenyon whereby it was noted that when CBD is administered in combination with other cancer treatment therapies, it promotes disruption intracellular signalling pathways, such as P13K/AKT/mTOR and ERK, and so enhances combinative treatment cell death outcomes [33].

Overall, these findings suggest that PDT treatment with FNBC coupled with CBD can successfully obliterate CRC cells due to enhanced PS bioavailability, AuNP-induced photothermal damage and CBD antiproliferative effect.

### Hoechst nuclear damage stain analysis

**Figure 11:**
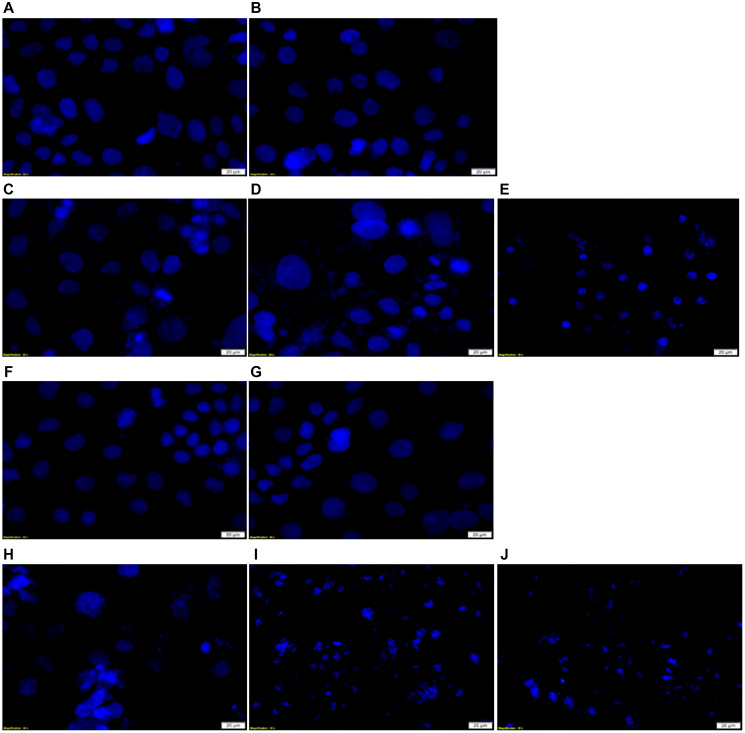
DNA damage Hoechst 33258 stained *in vitro* CRC cells examination in FNBC PDT response assays with and without CBD treatment. (**A**) Cells only, (**B**) Cells + Irradiation (10 J/cm^2^), (**C**) Cells + 0.125 μM ZnPcS_4_ + Irradiation, (**D**) Cells + 0.125 μM ZnPcS_4_ – AuNP-PEG5000-SH- NH_2_ + Irradiation, (**E**) Cells + 0.125 μM ZnPcS_4_ – AuNP-PEG5000-SH- NH_2_ – Anti-GCC Ab + Irradiation, (**F**) Cells + 1 uM CBD + Irradiation, (**G**) Cells + Ethanol, (**H**) Cells + 0.125 μM ZnPcS_4_ + 1 uM CBD + Irradiation, (**I**) Cells + 0.125 μM ZnPcS_4_ – AuNP-PEG5000-SH- NH_2_ + 1 uM CBD + Irradiation and (**J**) Cells + 0.125 μM ZnPcS_4_ – AuNP-PEG5000-SH- NH_2_ – Anti-GCC Ab + 1 uM CBD + Irradiation.

Hoechst 33258 stain was used to determine the degree of cellular nuclear DNA damage in various control and experimental groups by comparing nuclear morphological images to cells only control, which received no treatment (Figure 11). The results showed a prominent and round nuclei in cells only control, cells which received laser irradiation alone, and cells treated with ethanol alone. However, there was a slight fragmentation in irradiated cells treated with PS alone, suggesting that it induced insignificant DNA damage. These outcomes can be attributed to the fact that second generation PSs (e.g., ZnPcS_4_) are inherently hydrophobic and tend to aggregate under physiological conditions, which hamper PS cellular uptake and cytotoxic ROS yield [34]. In irradiated cells treated with ZnPcS_4_ PS-AuNP, irregular nuclear shrinkage was observed, with some nuclei becoming swollen and fragmented in shape, which suggests DNA damage. Studies by Manoto linked nuclear shrinkage in irradiated CRC cells treated with 10 μM ZnPcS_mix_ to signify apoptosis [35]. This improved DNA damage can be attributed to the fact that the ZnPcS_4_ PS was combined with AuNP, which increased cellular uptake and ROS yield [9]. Additionally, AuNPs enhance PDT overall efficacy by exerting photothermal destruction [9]. In comparison to the above discussed groups, irradiated cells treated with FNBC showed prominent nuclei shrinkage and fragmentation, suggesting late apoptotic cell death. This improved outcome can be attributed to fact that FNBC had a specific Ab for CRC, which increased intracellular accumulation and active localization of PS in CRC cells, with additional photothermal destruction of AuNP.

Irradiated cells treated with CBD presented with irregular nuclear shrinkage and some nuclei appeared irregularly condensed and fragmented in shape, which suggested apoptosis. These findings coincided with studies by Aviello which highlighted that CBD counteracted cellular proliferation via induction of apoptotic cell death pathways [19]. However, the highest degree of DNA damage was noted PDT treated experimental groups combined with CBD, particularly FNBC, whereby a complete nuclei and DNA destruction were observed. Studies by Balusamy noted when the cells display complete nuclear shrinkage and fragmentation, it indicates DNA conformation and chromatin state disruption, which is a hallmark of late apoptosis or necrosis [36].

## DISCUSSION

The FNBC consisting of ZnPcS_4_ PS, AuNP-PEG-SH-NH_2_ and Anti-GCC Ab was successfully synthesized for the PDT treatment of *in vitro* cultured CaCo-2 CRC cells. ZnPcS_4_ PS and AuNP formed a strong Au-S bond via ligand exchange, as well as weak disulphide bond occurred between them via ligand absorption process. Characterization techniques noted a well-oriented immobilization of Anti-GCC Abs onto the surface of amine functionalized AuNPs via C-terminus amide bonding without destruction of the Ab N-terminus binding site of the Ab to allow for effective tumor antigen recognition. DLS and ZP analysis concluded that FNBC was homogeneously pure, monodispersed, small in size and highly stable which certified it as an efficient ZnPcS_4_ PS nanocarrier. These results suggested that all the three individual constituents (ZnPcS_4_, AuNP-PEG5000-SH-NH_2_ and Anti-GCC Ab) were successfully bonded together to form one single molecule [21].

The FNBC significantly enhanced the intracellular accumulation of ZnPcS_4_ PS in CaCo-2 cells when compared to PS alone and PS-AuNPs. Overall, the functionalization of PS-AuNPs with Anti-GCC Abs further increased the PS’s active subcellular localization via GCC-receptor-mediated endocyotosis, and so under laser light irradiation at the wavelength of 673 nm proved to be a highly effective nanodrug for eradicating *in vitro* cultured CaCo-2. These findings coincided with studies conducted by Gao which reported that PSs when encapsulated into an active targeting moiety are more effectively taken up by CRC cells, which in turn exerted appreciable anti-proliferative effects within these cells [37].

Additionally, this study noted that the administration of CBD alone demonstrated a considerable inhibitory effect on CRC cells viability and the application of laser irradiation had a negligible effect. These results suggest that CBD is solely an effective chemo preventive agent. These findings are supported by a study performed by Kim, whereby *in vitro* cultured CRC cells treated with CBD, noted a significant decrease in cellular viability due to apoptotic cell death [38]. However, the most appreciably significant results were noted within PDT treated experimental groups which received the FNBC coupled with CBD treatment, whereby the cellular morphology of cells was severely disrupted and the majority of cells were non-viable, which concurred with reports by Hong stating that the modification of PSs with AuNPs, improves their passive uptake and their hydrophilicity via EPR effect [7]. Additionally, AuNPs tend to augment overall PDT-mediated cell destruction by exerting photothermal destruction [25]. Naidoo and colleagues also highlighted that when AuNP-PSs are incorporated with targeting entities (e.g., mAbs), PS passivation in cells is specifically enhanced and actively internalized, and so this form targeted PDT is most effective in cancer therapy [25].

Furthermore, the combined effect of this treatment with CBD demonstrated a far more improved nuclear DNA damage [36], suggestive that combinative PDT FNBC and CBD could possibly eradicate both primary and secondary tumors. Therefore, the results of irradiated FNBC combined with CBD concur with reports by Kenyon which stated that when CBD is coupled with other cancer therapies, it modulates intracellular signaling death pathways, which in turn improves combinative treatment efficacy [33]. In conclusion, based on the findings from this study, the hypothesized FNBC PDT active targeting therapy of *in vitro* CRC cells for the primary treatment of CRC is a plausible enhancement, however when this PDT therapy is combined with CBD for the eradication of resistant CRC stem like cells to prevent secondary systemic disease it is highly significant.

There is currently no data on the combined effect of CBD and targeted PDT treatment of CRC, the current study investigated the combination therapy in treating primary CRC tumours, as well as inhbiting the secondary malignant spread of CRC, since it has not been accomplished to date. Therefore, this study warrants further investigation into the therapeutic efficacy of this targeted PDT treatment, as well as its combinative treatment with CBD using models that can readily recapitulate human response. Virtually all *in vitro* PDT experiments are performed on monolayer cultures and the cellular environments of these models do not mimic that of human tumors [39]. Therefore, three-dimensional cell cultures models may serve as excellent models for evaluating the combined efficacy of CBD and PDT in cancer research since they mimic different features of solid tumours far more superior [40].

## MATERIALS AND METHODS

### Cell culture

CaCo-2 CRC cell line CaCo-2 (Cellonex Cat SS1402 CCAC-FL; CCAC-C) was commercially purchased. Cells were grown in Dulbecco’s Modified Eagle Medium (D5796, Sigma Aldrich) that supplemented with 4 mM sodium pyruvate, 4 mM L-glutamine, 2.5 g/ml amphotericin B, and 100 U Penicillin 100 g/ml streptomycin solution, as well as was enriched with 10% Foetal Bovine Serum. Cells were cultured in T175 culture flask in an in an 85% humidified condition at 37°C and 5% CO_2_. Cells were subculture upon reaching 80–90% confluency and seeded into culture dishes at density of 6 × 10^5^ cells/3 ml of supplemented media. These small cell cultures dishes were incubated for 4 h to allow cellular attachment before conducting experiments.

### Cell treatment and laser irradiation

Post 4 h incubation, culture dishes were divided into various control and experimental groups for CBD/ZnPcS_4_ PS PDT dose response assays or used in final FNBC alone with or without CBD PDT experiments. The old cell culture media was discarded and replaced. Then the groups to be treated with 1 μM CBD alone or in combination with the FNBC and its controls, which consisted of: 0.125 μM ZnPcS_4_, 0.125 μM ZnPcS_4_ + 3.39 × 10^12^ AuNP-PEG-SH-NH_2_ particles/ml, 0.125 μM ZnPcS_4_ + 0.11 μg/ml Anti-GCC Ab or FNBC (0.11 μg/ml Anti-GCC Ab, 3.39 × 10^12^ AuNP-PEG-SH-NH_2_ particles/ml and 0.125 μM of ZnPcS_4_ in 0.001 M PBS) had these concentrations added to their media and were re-incubated for 20 h. Then PDT groups, as well as the laser irradiation control and experimental groups were irradiated for 16 min and 8 sec using a Roithner 1000 mA 673 nm high power semiconductor diode laser (Arroyo 4210) at a fluency of 10 J/cm^2^. After irradiation the culture media was removed and replaced in all the culture dishes, and they were then incubated for an additional 24 h.

### ZnPcS_4_ photosensitizer dose response assays

Stock solution of 0.0005 M ZnPcS_4_ PS (Santa Cruz^®^ Biotechnology sc-264509A) was diluted to make a working concentration of 0.000125 M or 125 μM in 4 ml 0.001 M phosphate buffered saline (PBS) (%w/v), which used throughout the experimentation. Varying concentrations of ZnPcS_4_ PS were dispensed to the cultured CRC cells. The lowest inhibitory concentration (ICD_50_) that was found to induce 50% cytotoxicity post-PDT (ICD_50_) was 0.125 μM. A 0.4% Trypan Blue Solution (Sigma Aldrich: T8154) stain was used to assess cell viability and inadvertently cellular cytotoxicity. Cell counts were determined using an automated cell counter Countess^®^ II FL (Invitrogen).

### Cannabidiol (CBD) dose response assays

10 mg/ml of Cannabidiol solution (CBD) solubilized in 1 ml 99.8% ethanol with a molecular mass of 314.46 g/mol, was commercially obtained from Sigma Aldrich (90899-1 ml). Upon receipt, CBD was further diluted with 19 ml of 99.8% of ethanol to make a stock concentration of 0.5 mg/ml. Varying doses of CBD were administered to CRC cells and the ICD_50_ was found to be 1 μM 24 h post irradiation. A 0.4% Trypan Blue Solution (Sigma Aldrich: T8154) stain was used to assess cell viability and inadvertently cellular cytotoxicity. Cell counts were determined using an automated cell counter Countess^®^ II FL (Invitrogen).

### Chemical synthesis and molecular characterization of the final nanobioconjugate (ZnPcS_4_ – AuNP – Anti-GCC)

Using methods adapted from Naidoo, Anti-GCC Ab (Abcam: ab122404) and working concentration ZnPcS_4_ PS were conjugated onto the surface of AuNP-PEG-SH-NH_2_ (Sigma-Aldrich: 765309) which contained 2.85 × 10^15^ AuNPs per ml (Sigma-Aldrich 765309) [4]. In brief 1 ml of AuNP-PEG-SH-NH_2_ (Sigma-Aldrich: 765309) was added to 1 ml of 125 μM ZnPcS_4_ in 4 ml 0.001 M PBS (%w/v). In order to promote spontaneous ligand exchange (between Au and PS tetra sulphides) and adsorption (disulphide bond formation between PEG and PS), this solution was vortexed overnight at room temperature. The solution was then micro centrifuged at 15 200 rpm for 1 hour to allow for purification. The supernatant was discarded and the pellet (which contained conjugated ZnPcS_4_ and AuNP-PEG-SH-NH_2_) was re-suspended in 1 ml PBS.

Covalent mode carbodiimide crosslinker two-step coupling EDC and NHS chemistry was used to activate C-terminus succinimidyl ester terminus on 200 μg/ml of Anti-GCC Ab (Abcam: ab122404). This activation allowed the Anti-GCC Ab to react with the amine group (NH_2_) on the AuNPs, which were already previously bound to the ZnPcS_4_ PS, and so when mixed together, formed a stable amide bond. This technique ensured that the bio-targeting antibody was correctly orientated i.e.: the N-terminus remained freely available for active targeting, while the C-terminus stalk was firmly bound to the amine functionalized AuNPs.

The FNBC (ZnPcS_4_–AuNP-PEG-SH-NH_2_–Anti-GCC Ab) then underwent various molecular characterization assays: UV-Visible and FT-IR Spectroscopy, DLS and ZP, as well as subcellular localization and uptake confirmation immunofluorescent staining assays, as described below.

### UV-Visible spectroscopy

The successful binding of the 3 chemical components within the FNBC (ZnPcS_4_–AuNP-PEG-SH-NH_2_–Anti-GCC Ab) was confirmed using Jenway Genova Nano Plus Life Science Spectrophotometer. The spectral analysis was performed using the purity scan mode (198 to 800 nm) and protein direct UV option (220 nm). The amount of Ab and ZnPcS_4_ PS, as well as number of bound AuNPs within the FNBC was confirmed by comparing initial spectra of the 3 unbound chemical components at known concentrations with final spectra obtained from the FNBC [25].

### Fourier transform infrared (FTIR) spectroscopy

The control AuNPPEG-SH-NH_2_–ZnPcS_4_ conjugate underwent FTIR analysis using PerkinElmer FTIR spectrometer to confirm the existence of strong Au-S and weak disulphide bonds via ligand exchange and absorption processes when compared to AuNPPEG-SH-NH_2_ alone. Furthermore, the FNBC also underwent FTIR analysis to confirm the formation of amide bonds in comparison to AuNPPEG-SH-NH_2_ FTIR spectra alone. The infrared spectra results were read at 400 to 4000 cm^–1^ frequency range with 25 scans.

### Dynamic light scattering (DLS) and zeta potential (ZP)

DLS and ZP analysis were performed in triplicate with 15 runs per run, using the Malvern Zetasizer Nano ZS (Malvern Instruments, Malvern UK). Twelve microliters of each sample were loaded into a clean, scratch free plastic Zeta 3 × 3 mm dip cell cuvette, which had in-built electrodes capable of DLS and Zeta measurements. All analyzed samples were heterogeneous or homogenous 10 to 50 μg/ml diluted suspensions in water, which were free from any form of precipitation. All experiments were performed at 25°C, at a 13° and 173° angle. Samples analyzed and compared consisted of AuNP-PEG-SH-NH_2_, ZnPcS_4_, ZnPcS_4_–AuNP-PEG-SH-NH_2_, Anti-GCC Ab and ZnPcS_4_–AuNP-PEG-SH-NH_2_–Anti-GCC Ab.

### Subcellular localization

CaCo-2 cells were seeded in a small culture dishes, which contained a coverslip. Culture dishes were divided into various control and experimental groups, which received treatments as discussed above. After 24 h of incubation, the cells were placed on ice and stained for 30 min with 2 μg/ml ICAM-1 mouse monoclonal IgG1 (AbAB2213 AC: Abcam) and 5 μg/ml Goat anti-mouse IgG-FITC (AB6785 AC: Abcam). Thereafter, the cells were stained at room temperature for 5 minutes with 1 μg/ml DAPI. The dishes were then rinsed with HBSS and the coverslips were mounted onto slides. The slides were examined using a Carl Zeiss Axio Z1 Observer fluorescent microscope, with various filter settings.

### Morphological assessment by light microscopy

Morphological changes in CaCo-2 cells were observed using an inverted microscope (Olympus CKX41, C5060-ADUS) which had a built-in digital camera at 100× magnification.

### Flow cytometry: cell viability

The Annexin V-FITC detection kit (BD Scientific: BD/556570), was used to determine the cell viability of the various control and experimental groups post incubation by means of a BD Accuri™ C6 flow cytometer.

### Hoechst nuclear damage stain

CaCo-2 cells were seeded in a small culture dishes, which contained a coverslip. Culture dishes were divided into various control and experimental groups, which received treatments as discussed above. After 24 h of incubation, the cells were stained with 1 μg/mL Hoechst 33258 Pentahydrate (bis-benzimide) (Thermofisher: H211491) for 15 min in the dark, at room temperature. Thereafter, the dishes rinsed with PBS and the coverslips were mounted onto slides. The slides were examined using a Carl Zeiss Axio Z1 Observer fluorescent microscope, with various filter settings.

### Statistics

All experiments were performed six times with duplicate assays runs and reported results correspond to these experiments mean and the standard deviation. Normal distributed data was analysed using one way analysis of variances and the students *t*-test. Non-normally distributed data was assessed using the Mann-Whitney test. Significance differences between the various control and experimental groups (*P* < 0.05^*^, *P* < 0.01^**^ or *P* < 0.001^***^) were accepted as statistically different, whereby values were reported in the 95% confidence interval.

## Abbreviations

Ab: Antibody
Anti-GCC: Anti-Guanylate Cyclase
AuNPs: Gold nanoparticles
CBD: Cannabidiol
CRC: Colorectal Cancer
CSCs: Colorectal Stem Cells
FNBC: Final Nanobioconjugate
PDT: Photodynamic Therapy
PS: Photosensitizer
ROS: Reactive Oxygen Species
ZnPc: Zinc Phthalocyanine
ZnPcS_mix_: Zinc Phthalocyanine Sulfonated mix
ZnPcS_4_: Zinc Phthalocyanine Tetrasulfonic Acid

## References

[1] Viswanath B , Kim S , Lee K . Recent insights into nanotechnology development for detection and treatment of colorectal cancer. Int J Nanomedicine. 2016; 11:2491–504. 10.2147/IJN.S108715. 27330292 PMC4898029

[2] Mármol I , Sánchez-de-Diego C , Jiménez-Moreno N , Ancín-Azpilicueta C , Rodríguez-Yoldi MJ . Therapeutic Applications of Rose Hips from Different Rosa Species. Int J Mol Sci. 2017; 18:1137. 10.3390/ijms18061137. 28587101 PMC5485961

[3] Nkune NW , Kruger CA , Abrahamse H . Possible Enhancement of Photodynamic Therapy (PDT) Colorectal Cancer Treatment when Combined with Cannabidiol. Anticancer Agents Med Chem. 2021; 21:137–48. 10.2174/1871520620666200415102321. 32294046

[4] Crous A , Abrahamse H . Effective Gold Nanoparticle-Antibody-Mediated Drug Delivery for Photodynamic Therapy of Lung Cancer Stem Cells. Int J Mol Sci. 2020; 21:3742. 10.3390/ijms21113742. 32466428 PMC7311980

[5] Kwiatkowski S , Knap B , Przystupski D , Saczko J , Kędzierska E , Knap-Czop K , Kotlińska J , Michel O , Kotowski K , Kulbacka J . Photodynamic therapy - mechanisms, photosensitizers and combinations. Biomed Pharmacother. 2018; 106:1098–107. 10.1016/j.biopha.2018.07.049. 30119176

[6] Kruger CA , Abrahamse H . Targeted Photodynamic Therapy as Potential Treatment Modality for the Eradication of Colon Cancer. Multidisciplinary Approach for Colorectal Cancer. 2019. 10.5772/intechopen.84760. 28990490

[7] Hong EJ , Choi DG , Shim MS . Targeted and effective photodynamic therapy for cancer using functionalized nanomaterials. Acta Pharm Sin B. 2016; 6:297–307. 10.1016/j.apsb.2016.01.007. 27471670 PMC4951583

[8] Mokwena MG , Kruger CA , Ivan MT , Heidi A . A review of nanoparticle photosensitizer drug delivery uptake systems for photodynamic treatment of lung cancer. Photodiagnosis Photodyn Ther. 2018; 22:147–54. 10.1016/j.pdpdt.2018.03.006. 29588217

[9] Kruger CA , Abrahamse H . Utilisation of Targeted Nanoparticle Photosensitiser Drug Delivery Systems for the Enhancement of Photodynamic Therapy. Molecules. 2018; 23:2628. 10.3390/molecules23102628. 30322132 PMC6222717

[10] Montaseri H , Kruger CA , Abrahamse H . Targeted Photodynamic Therapy Using Alloyed Nanoparticle-Conjugated 5-Aminolevulinic Acid for Breast Cancer. Pharmaceutics. 2021; 13:1375. 10.3390/pharmaceutics13091375. 34575450 PMC8471498

[11] Sekhejane PR , Houreld NN , Abrahamse H . Multiorganelle Localization of Metallated Phthalocyanine Photosensitizer in Colorectal Cancer Cells (DLD-1 and CaCo-2) Enhances Efficacy of Photodynamic Therapy. International Journal of Photoenergy. 2014; 2014:383027. 10.1155/2014/383027.

[12] Mfouo-Tynga IS , Dias LD , Inada NM , Kurachi C . Features of third generation photosensitizers used in anticancer photodynamic therapy: Review. Photodiagnosis Photodyn Ther. 2021; 34:102091. 10.1016/j.pdpdt.2020.102091. 33453423

[13] Danaee H , Kalebic T , Wyant T , Fassan M , Mescoli C , Gao F , Trepicchio WL , Rugge M . Consistent expression of guanylyl cyclase-C in primary and metastatic gastrointestinal cancers. PLoS One. 2017; 12:e0189953. 10.1371/journal.pone.0189953. 29261789 PMC5736218

[14] Wei MF , Chen MW , Chen KC , Lou PJ , Lin SY , Hung SC , Hsiao M , Yao CJ , Shieh MJ . Autophagy promotes resistance to photodynamic therapy-induced apoptosis selectively in colorectal cancer stem-like cells. Autophagy. 2014; 10:1179–92. 10.4161/auto.28679. 24905352 PMC4203546

[15] Shams M , Owczarczak B , Manderscheid-Kern P , Bellnier DA , Gollnick SO . Development of photodynamic therapy regimens that control primary tumor growth and inhibit secondary disease. Cancer Immunol Immunother. 2015; 64:287–97. 10.1007/s00262-014-1633-9. 25384911 PMC4341021

[16] Wu HY , Huang CH , Lin YH , Wang CC , Jan TR . Cannabidiol induced apoptosis in human monocytes through mitochondrial permeability transition pore-mediated ROS production. Free Radic Biol Med. 2018; 124:311–18. 10.1016/j.freeradbiomed.2018.06.023. 29940353

[17] Jeong S , Yun HK , Jeong YA , Jo MJ , Kang SH , Kim JL , Kim DY , Park SH , Kim BR , Na YJ , Lee SI , Kim HD , Kim DH , et al. Cannabidiol-induced apoptosis is mediated by activation of Noxa in human colorectal cancer cells. Cancer Lett. 2019; 447:12–23. 10.1016/j.canlet.2019.01.011. 30660647

[18] Lukhele ST , Motadi LR . Cannabidiol rather than Cannabis sativa extracts inhibit cell growth and induce apoptosis in cervical cancer cells. BMC Complement Altern Med. 2016; 16:335. 10.1186/s12906-016-1280-0. 27586579 PMC5009497

[19] Aviello G , Romano B , Borrelli F , Capasso R , Gallo L , Piscitelli F , Di Marzo V , Izzo AA . Chemopreventive effect of the non-psychotropic phytocannabinoid cannabidiol on experimental colon cancer. J Mol Med (Berl). 2012; 90:925–34. 10.1007/s00109-011-0856-x. 22231745

[20] Yu X , Trase I , Ren M , Duval K , Guo X , Chen Z . Design of Nanoparticle-Based Carriers for Targeted Drug Delivery. J Nanomater. 2016; 2016:1087250. 10.1155/2016/1087250. 27398083 PMC4936496

[21] Bhattacharjee S . DLS and zeta potential - What they are and what they are not? J Control Release. 2016; 235:337–51. 10.1016/j.jconrel.2016.06.017. 27297779

[22] Honary S , Zahir F . Effect of Zeta Potential on the Properties of Nano-Drug Delivery Systems - A Review (Part 1). Trop J Pharm Res. 2013; 12:255–64. 10.4314/tjpr.v12i2.19.

[23] Brozek-Pluska B , Orlikowski M , Abramczyk H . Phthalocyanines: From Dyes to Photosensitizers in Diagnostics and Treatment of Cancer. Spectroscopy and Raman Imaging Studies of Phthalocyanines in Human Breast Tissues. Handbook of Porphyrin Science. 2016; 1–49. 10.1142/9789813149595_0001.

[24] Yao C , Zhang L , Wang J , He Y , Xin J , Wang S , Xu H , Zhang Z . Gold Nanoparticle Mediated Phototherapy for Cancer. Journal of Nanomaterials. 2016; 2016:5497136. 10.1155/2016/5497136.

[25] Naidoo C , Kruger CA , Abrahamse H . Targeted photodynamic therapy treatment of *in vitro* A375 metastatic melanoma cells. Oncotarget. 2019; 10:6079–95. 10.18632/oncotarget.27221. 31692760 PMC6817449

[26] Hermanson G . Nanomaterial Bioconjugation Techniques. Available online: https://www.sigmaaldrich.com/materials-science/nanomaterials/nanomaterial-bioconjugation-techniques.html (accessed on 9 October 2021).

[27] Ekka D , Roy MN . Quantitative and qualitative analysis of ionic solvation of individual ions of imidazolium based ionic liquids in significant solution systems by conductance and FT-IR spectroscopy. RSC Advances. 2014; 4:19831–45. 10.1039/C3RA48051H.

[28] Nguyen KC . Quantitative analysis of COOH-terminated alkanethiol SAMs on gold nanoparticle surfaces. Advances in Natural Sciences: Nanoscience and Nanotechnology. 2012; 3:045008. 10.1088/2043-6262/3/4/045008.

[29] Mecozzi M , Sturchio E . Computer Assisted Examination of Infrared and Near Infrared Spectra to Assess Structural and Molecular Changes in Biological Samples Exposed to Pollutants: A Case of Study. J Imaging. 2017; 3:11. 10.3390/jimaging3010011.

[30] Wu B , Zhao N . A Targeted Nanoprobe Based on Carbon Nanotubes-Natural Biopolymer Chitosan Composites. Nanomaterials (Basel). 2016; 6:216. 10.3390/nano6110216. 28335344 PMC5245750

[31] Honarmand M , Namazi F , Mohammadi A , Nazifi S . Can cannabidiol inhibit angiogenesis in colon cancer? Comp Clin Pathol. 2019; 28:165–72. 10.1007/s00580-018-2810-6.

[32] Raup-Konsavage WM , Johnson M , Legare CA , Yochum GS , Morgan DJ , Vrana KE . Synthetic Cannabinoid Activity Against Colorectal Cancer Cells. Cannabis Cannabinoid Res. 2018; 3:272–81. 10.1089/can.2018.0065. 30671539 PMC6340378

[33] Kenyon J , Liu W , Dalgleish A . Report of Objective Clinical Responses of Cancer Patients to Pharmaceutical-grade Synthetic Cannabidiol. Anticancer Res. 2018; 38:5831–35. 10.21873/anticanres.12924. 30275207

[34] Hodgkinson N , Kruger CA , Abrahamse H . Targeted photodynamic therapy as potential treatment modality for the eradication of colon cancer and colon cancer stem cells. Tumour Biol. 2017; 39:1010428317734691. 10.1177/1010428317734691. 28990490

[35] Manoto SL , Sekhejane PR , Houreld NN , Abrahamse H . Localization and phototoxic effect of zinc sulfophthalocyanine photosensitizer in human colon (DLD-1) and lung (A549) carcinoma cells (*in vitro*). Photodiagnosis Photodyn Ther. 2012; 9:52–59. 10.1016/j.pdpdt.2011.08.006. 22369729

[36] Balusamy SR , Perumalsamy H , Huq MA , Balasubramanian B . Anti-proliferative activity of Origanum vulgare inhibited lipogenesis and induced mitochondrial mediated apoptosis in human stomach cancer cell lines. Biomed Pharmacother. 2018; 108:1835–44. 10.1016/j.biopha.2018.10.028. 30372889

[37] Gao S , Wang J , Tian R , Wang G , Zhang L , Li Y , Li L , Ma Q , Zhu L . Construction and Evaluation of a Targeted Hyaluronic Acid Nanoparticle/Photosensitizer Complex for Cancer Photodynamic Therapy. ACS Appl Mater Interfaces. 2017; 9:32509–19. 10.1021/acsami.7b09331. 28875691

[38] Kim JL , Kim BR , Kim DY , Jeong YA , Jeong S , Na YJ , Park SH , Yun HK , Jo MJ , Kim BG , Kim HD , Kim DH , Oh SC , et al. Cannabidiol Enhances the Therapeutic Effects of TRAIL by Upregulating DR5 in Colorectal Cancer. Cancers (Basel). 2019; 11:642. 10.3390/cancers11050642. 31075907 PMC6562873

[39] Costa EC , Moreira AF , de Melo-Diogo D , Gaspar VM , Carvalho MP , Correia IJ . 3D tumor spheroids: an overview on the tools and techniques used for their analysis. Biotechnol Adv. 2016; 34:1427–41. 10.1016/j.biotechadv.2016.11.002. 27845258

[40] Nkune NW , Abrahamse H . Nanoparticle-Based Drug Delivery Systems for Photodynamic Therapy of Metastatic Melanoma: A Review. Int J Mol Sci. 2021; 22:12549. 10.3390/ijms222212549. 34830431 PMC8620728

